# Abnormal DNA content in oral epithelial dysplasia is associated with increased risk of progression to carcinoma

**DOI:** 10.1038/sj.bjc.6605905

**Published:** 2010-09-21

**Authors:** G Bradley, E W Odell, S Raphael, J Ho, L W Le, S Benchimol, S Kamel-Reid

**Affiliations:** 1 Faculty of Dentistry, University of Toronto, 124 Edward Street, Toronto, Ontario, Canada M5G 1G6; 2 Ontario Cancer Institute, University Health Network, University of Toronto, 610 University Avenue, Toronto, Ontario, Canada M5G 2M9; 3 Department of Oral Pathology, King's College London, Floor 28 Tower, Guy's Hospital, London, SE1 9RT, UK; 4 Department of Pathology, Sunnybrook Health Sciences Centre, University of Toronto, Bayview Avenue, Toronto, Ontario, Canada M4N 3N5; 5 Applied Molecular Profiling Laboratory, Ontario Cancer Institute, University of Toronto, Toronto, Ontario, Canada; 6 Department of Biostatistics, University Health Network, Toronto, Ontario, Canada; 7 Department of Biology, York University, 4700 Keele Street, Toronto, Ontario, Canada M3J 1P3; 8 Department of Pathology, University Health Network, Toronto, Ontario Canada

**Keywords:** DNA cytometry, oral, epithelial dysplasia, carcinoma

## Abstract

**Background:**

Oral epithelial dysplasia (OED) is a histologically detectable lesion that may progress to carcinoma but there are no accurate markers that predict progression. This study examined the development of carcinoma from oral dysplastic lesions, and the association between abnormal DNA content and progression to carcinoma.

**Methods:**

Epithelial dysplasias from the Oral Pathology Diagnostic Service were matched against the Ontario Cancer Registry database to identify cases that progressed to carcinoma. A case–control study was conducted to compare DNA image cytometry of dysplasias that progressed with those that have not progressed. For a subset of the progressed dysplasias, DNA content of the carcinoma was also analysed.

**Results:**

A total of 8% of epithelial dysplasias progressed to carcinoma after 6–131 months. In all, 28 of 99 dysplasias showed abnormal DNA content by image cytometry. In multivariate analysis of time to progression, abnormal DNA content was a significant predictor with hazard ratio of 3.3 (95% confidence interval: 1.5–7.4) corrected for site and grade of dysplasia. Analysis of sequential samples of dysplasia and carcinoma suggested that epithelial cell populations with grossly abnormal DNA content were transient intermediates during oral cancer development.

**Conclusions:**

Abnormal DNA content is a significant biomarker of a subset of OED that progress to carcinoma.

Oral squamous carcinoma develops through multiple steps of accumulation of genetic and epigenetic alterations, which implies that elimination of pre-invasive, precursor lesions can reduce the morbidity and mortality of this malignant disease (Califano *et al*, 1996; Reibel, 2003). The best-known precursor lesion is epithelial dysplasia, which is histologically detectable and often presents clinically as white or red mucosal patches called leukoplakia and erythroplakia (Warnakulasuriya *et al*, 2007). However, only a minority of epithelial dysplasias progress to carcinoma, whereas other dysplastic lesions remain unchanged for years or resolve over time (Napier and Speight, 2008). Currently, assessment of risk of carcinoma is based on clinical and histological features, but there is no available marker that can accurately predict the behaviour of an individual dysplastic lesion so that appropriate treatment can be rendered to prevent carcinoma (Reibel, 2003). Histological grading is subjective, with intra- and inter-observer variability (Warnakulasuriya *et al*, 2008). Computer-assisted analysis of histological appearance has been reported to increase prognostic value but it still relies on morphological changes to predict behaviour of the lesion (Guillaud *et al*, 2008).

Several molecular aberrations have been studied as predictive markers for progression of oral epithelial dysplasia (OED) to carcinoma. These include immunohistochemical staining for the p53 tumour suppressor protein (Cruz *et al*, 1998), overexpression of matrix metalloproteinase mRNA (Jordan *et al*, 2004), loss of heterozygosity (LOH) at specific chromosomal loci (Mao *et al*, 1996; Rosin *et al*, 2000), promoter methylation of the p16 tumour suppressor gene (Hall *et al*, 2008) and alterations in microRNA expression (Cervigne *et al*, 2009). These markers have not yet been widely adopted for assessment of risk of progression of OED.

The development of oral squamous carcinoma is a complex multistep process that may involve escape from senescence, loss of p53 and p16 function, and genetic instability (Hunter *et al*, 2005). We hypothesise that changes in cellular DNA content, or DNA aneuploidy, is a critical event during oral squamous carcinoma development. Studies of cervical carcinogenesis and Barrett's oesophagus have shown that genetic instability occurred during malignant progression (Galipeau *et al*, 1996; Rabinovitch *et al*, 2001; Olaharski *et al*, 2006). A recent study of OED showed that DNA aneuploidy was more frequent among dysplastic lesions that progressed than among lesions that did not progress (Torres-Rendon *et al*, 2009).

In this study, we reviewed a large series of OED from an outpatient oral biopsy service and identified the cases that progressed from dysplasia to carcinoma by linkage of the biopsy database with the cancer registry. A retrospective case–control study was conducted to study the association between abnormal DNA content and the clinical outcome of whether the lesion progressed to carcinoma, or not progressed after a reasonable follow-up period. We showed that DNA content analysis by image cytometry can be successfully applied to most archival, formalin-fixed, paraffin-embedded tissue specimens. Distinctive patterns of DNA content distribution could be recognised and provided information of biological and clinical significance.

## Materials and methods

### Collection of dysplasia and carcinoma cases

This retrospective study has been approved by the Research Ethics Boards of the University of Toronto (protocol reference number 21571) and Sunnybrook Health Sciences Centre (project identification number 363–2008). The files of the Oral Pathology Diagnostic Service (OPDS) at the Faculty of Dentistry, University of Toronto, were searched for all cases coded as epithelial dysplasia for the years 1993–2002 inclusive. The list of dysplasia was matched against the oral cancer database of the Ontario Cancer Registry, which collects data on all cases of cancer diagnosed in the province. The match produced a sub-list of cases of OED that were associated with subsequent carcinoma at the same anatomical site or a contiguous site. Examples of contiguous sites included tongue and floor of the mouth, and mandibular mucosa and floor of the mouth. The majority of the diagnoses of oral carcinoma were made in different hospitals across the province, including Sunnybrook Health Science Centre (SHSC). In a small number of cases, the oral carcinoma diagnosis was made at the OPDS at the University of Toronto. We were able to retrieve the paraffin-embedded samples of oral carcinoma from SHSC and OPDS for analyses in conjunction with the preceding dysplastic lesion.

Cases of OED that did not produce a match from the oral cancer database would include epithelial dysplasias that have not progressed to oral cancer. It was also possible that the patient has moved out of province and developed oral carcinoma elsewhere. Therefore, we sought confirmation that the patient has not moved out of province and was alive with no evidence of oral cancer, from the clinician who submitted the biopsy of the dysplastic lesion. We identified a group of ‘control’ patients whose dysplastic lesion has not progressed after a minimum follow-up period of 5 years. The control group was chosen to match the group of patients with ‘progressed’ dysplasia for age and sex. Five cases of oral squamous papilloma, which is a hyperplastic lesion of stratified squamous epithelium, were taken from the OPDS files and used as reference cases for DNA content analysis.

### Analysis of DNA content

**Figure 1:**
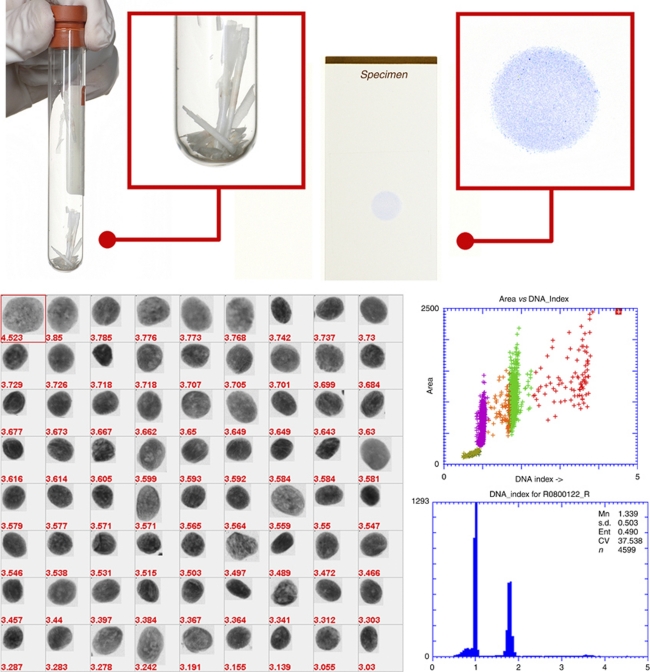
DNA content analysis of biopsy specimens. Top panel, left: 50-*μ*m thick paraffin sections before de-paraffinisation and rehydration; right: cytospin preparation of nuclei after Feulgen–thionin staining. The bottom panel shows an example of DNA content analysis of a dysplasia sample with abnormal DNA content; left: image gallery of aneuploid nuclei (DNA index (DI) ⩾2.3), showing that each event with abnormal DNA content corresponds with a single nucleus and is not an artefact of clumped nuclei or debris; right: scatter plot of area of nucleus and DI, where nuclei were colour-coded according to DI, shown above the corresponding DNA histogram. CV, coefficient of variation; Ent, entropy of histogram data; Mn, mean.

*Isolation of nuclei from paraffin-embedded tissue* Between three and six thick sections (50 *μ*m) were cut from each paraffin block, depending on the size of tissue in the block. Thick sections were needed to avoid partial nuclei (Hedley, 1994). If necessary, areas of skeletal muscle or submucosal tissue were trimmed away from the paraffin block before sections were cut. Sections were de-paraffinised in xylene and rehydrated in graded ethanol to distilled water. The rehydrated tissues were digested with type XXIV proteinase (2 ml at 0.5 mg ml^–1^ in PBS, P8038; Sigma-Aldrich, Oakville, ON, Canada) in a 37°C water bath for 90 min with vigorous shaking. Digestion was stopped by adding an equal volume of cold PBS, and undigested tissue was separated from the suspension of nuclei by filtering through a 70 *μ*m mesh (BD Falcon 352350, VWR Mississauga, ON, Canada). The nuclei were gently spun down by centrifugation and the pellet was resuspended in cold PBS (Diwakar *et al*, 2005). The concentration of nuclei was estimated by counting a sample in a hemocytometer and adjusted to ∼300–400 nuclei per *μ*l. A monolayer of the nuclei was made by cytospin (Thermo Shandon Cytospin 4 cytocentrifuge, Fisher Scientific, Ottawa, ON, Canada), air-dried and sent for DNA staining and image cytometry (Figure 1).

*DNA image cytometry* Image cytometric analysis for DNA content of samples of dysplasia and carcinoma were performed by an accredited quantitative cytology laboratory (Perceptronix Medical Inc, Vancouver, BC, Canada). Cases of oral squamous papilloma from the OPDS were used as reference. All samples were analysed by laboratory personnel with no knowledge of the diagnoses. The cytospin preparations of nuclei were stained by the Feulgen–thionin method of stochiometric DNA staining (Clear2C staining kit, Perceptronix Medical Inc). Image cytometric analysis was performed using an automated image cytometer (registered *in vitro* diagnostic medical device ClearCyte, Perceptronix Medical Inc (Health Canada License Number 7062; CE Mark Registration Number IE/CA01/R/IV/0773/5048)) that was operated within the requirements given in the ESACP consensus report (Haroske *et al*, 2001). The image cytometer consisted of the following components: microscope with automated programmable XYZ stage and controller (AxioPlan2, Carl Zeiss Jena GmbH, Thornwood, NY, USA), automated programmable slide loader (Ludl Electronic Products Ltd, Hawthorne, NY, USA), high-resolution monochrome CCD camera (QICAM, Qimaging, Vancouver, BC, Canada), computer with high-resolution monitor and image analysis software. The system used a × 20 objective (numerical aperture 0.75) with a × 0.63 projection lens, resulting in spatial resolution of 0.54 *μ*m and effective pixel size of 0.37 × 0.37 *μ*m^2^. The image analysis software calculated features characterising the size, shape and distribution of optical density within the objects (Doudkine *et al*, 1995). These features were used by classification algorithms trained to recognise isolated epithelial nuclei and leukocytes from debris, overlapping cells and objects out of focus. Normal epithelial cells present within each sample were used as internal control. The DNA index (DI) (normalised DNA content) was used to classify epithelial nuclei into four groups: DI of 0.85–1.15, DI of 1.15–1.7, DI of 1.7–2.3 and DI >2.3, corresponding to diploid nuclei in G0/G1, S phase, G2/M phase or tetraploid nuclei, and aneuploid nuclei, respectively. A gallery of imaged objects was presented for visual review by a cytopathologist who confirmed that the right population of cells was used for normalisation and that the coefficient of variation of the diploid peak was <5%, and then verified objects in the non-diploid groups. A frequency histogram of DI and a report of the analysis were generated for each sample (Figure 1). Typically, 1000–5000 epithelial nuclei could be analysed in a cytospin preparation. A small number of cases with <500 nuclei yielded histograms of poor quality and were excluded from further analyses.

*Classification of DNA content/criteria for abnormal DNA histogram* The criteria for classification of DNA content as ‘abnormal’ or ‘no abnormality detected’ were based on published studies of OED and carcinoma (Diwakar *et al*, 2005; Klanrit *et al*, 2007; Torres-Rendon *et al*, 2009). Samples were considered to have no detectable abnormality if the histogram showed one peak of nuclei with DI of 0.85–1.15 (diploid nuclei in G0 or G1), fewer than 1% of nuclei had DI >2.3, and there were fewer than 10% of the nuclei with DI of 1.7–2.3 (nuclei in G2/M). All five cases of squamous papilloma clearly fit these three criteria.

### Immunohistochemical staining

Sections of 4 *μ*m thickness were cut onto coated slides (number 00211, Surgipath, Winnipeg, MB, Canada). Sections were de-paraffinised in xylene, and hydrated in graded ethanol to distilled water. Endogenous peroxidase was blocked by treatment with 3% hydrogen peroxide in distilled water for 10 min. For antigen retrieval, slides were immersed and heated in 10 mM citrate buffer (pH 6) for 10 min at 120°C in a pressurised chamber (Milestone Srl, Esbe Scientific, Toronto, ON, Canada) and then allowed to cool for 10 min. Sections were incubated with 5% normal horse serum in TBST (Tris-buffered saline with 0.1% Tween) for 30 min, followed by incubation at 4°C overnight with monoclonal antibody to p53 (clone DO-7, DAKO M7001, Mississauga, ON, Canada) at 1/100 dilution in normal serum-TBST. The primary antibody was followed by biotinylated horse anti-mouse IgG and avidin–biotin–peroxidase conjugate (Vectastain Elite ABC Kit, Vector Labs, Burlington, ON, Canada). Each incubation was followed by washes in TBST. The target antigen was visualised by treatment with Nova Red substrate solution (Vector Labs). Hematoxylin was used as counterstain. Staining was assessed on a semiquantitative scale: 0, no staining; 1, weak staining in <10% of epithelial nuclei; 2, moderate/strong staining in 10–50% of epithelial nuclei; 3, moderate/strong staining in >50% of epithelial nuclei.

### Statistical analyses

Statistical analyses were performed with SAS (version 9.1, SAS Institute, Cary, NC, USA). Data were summarised using descriptive statistics. The exact Cochran–Armitage trend test was performed to investigate the association between histological grade of dysplasia and finding of concurrent or subsequent carcinoma and the association between grade of dysplasia and abnormal DNA content. The proportions of abnormal DNA content among three site groups were compared using Fisher's exact test. Our primary outcome is time to progression to carcinoma from diagnosis of dysplasia. Time to progression to carcinoma for dysplastic lesions with or without abnormality of DNA content was examined in Kaplan–Meier plots. Log-rank test was used to compare the time to progression between the two groups. Time to progression to carcinoma for lesions with different grades of dysplasia was also examined similarly. Multivariate analysis of time to progression was performed using Cox proportional hazard model with abnormal DNA content, grade of dysplasia and site included as predictors in the model. Differences were considered statistically significant if *P*<0.05.

## Results

### Progression of dysplasia to carcinoma

A search of the files of the OPDS for the years 1993–2002 inclusive for cases diagnosed as epithelial dysplasia yielded 1477 biopsies from 1313 patients. The distribution of the biopsies by grade of dysplasia and oral site are shown in Table 1. A match of the list of dysplasia against the Ontario Cancer Registry for Oral Cancer led to identification of 114 cases of dysplasia from 82 patients that were followed by verrucous carcinoma or squamous cell carcinoma at the same anatomical site or a contiguous site, with an interval of at least 6 months between dysplasia and carcinoma. These cases were considered as progression of dysplasia to carcinoma. In addition, there were 44 cases of dysplasia from 40 patients that were associated with carcinoma at the same anatomical site within 6 months, considered as dysplasia concurrent with carcinoma. The finding of subsequent carcinoma and concurrent carcinoma by histological grade of dysplasia and by oral site are shown in Table 1. There was a significant association between grade of dysplasia and occurrence of carcinoma (either subsequent or concurrent), with *P*<0.0001 using Cochran–Armitage trend test.

The interval between dysplasia and carcinoma for the 82 progressed patients varied from 6 months to 131 months with a median of 44 months. For patients who had multiple biopsies of dysplasia preceding carcinoma, the interval was calculated from the latest dysplasia before carcinoma. For 59 of the 82 patients (72%) who progressed, carcinoma developed within 5 years of the latest dysplasia.

### DNA content analysis of dysplasia samples

*Selection of ‘progressed’ and ‘have not progressed’ samples for DNA content analysis* Among the 82 patients who progressed from dysplasia to carcinoma, there were 49 patients for whom a paraffin block of dysplasia could be retrieved from storage, with sufficient tissue for successful analysis of DNA content. The reasons for inability to analyse DNA content included tissue depleted in previous research projects, block unavailable because it was returned to the primary institution, and insufficient tissue in the block to yield enough epithelial nuclei for DNA analysis.

Among patients who did not match the Oral Cancer database, we identified 50 patients for whom we were able to confirm with the clinician who submitted the biopsy that the patient was alive with no evidence of oral cancer after a follow-up period of 5 years or more. The requirement of at least 5 years of follow-up was based on the finding that the majority of patients with progressed dysplasias developed carcinoma within 5 years of the dysplasia.

Table 2 shows the characteristics of the two patient groups for whom DNA content analysis was carried out.

**Figure 2:**
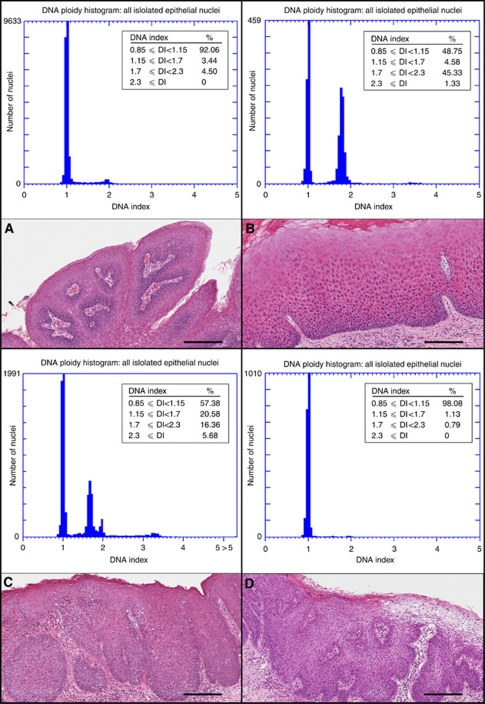
DNA histogram and haematoxylin–eosin stained section for representative cases. (**A**) Squamous papilloma used as reference in this study. No abnormality was detected in the DNA histogram. The large peak consisted of nuclei in G0/G1 phases of cell cycle and the small peak consisted of nuclei in G2/M phases. (**B**) Moderate dysplasia of the tongue that progressed to carcinoma after 41 months. Altogether, 45% of nuclei had a DNA index (DI) between 1.7 and 2.3 (peritetraploid peak) and 1.3% of the nuclei were aneuploid (DI ⩾2.3). (**C**) Severe dysplasia of the floor of the mouth that progressed to carcinoma after 6 months. There was an abnormal peak with DI between 1.15 and 1.7 (21% of nuclei, hyperdiploid peak), 16% of nuclei in the peritetraploid region and 5.7% of nuclei were aneuploid. (**D**) Carcinoma *in situ* of the floor of mouth that progressed to carcinoma in 61 months. No abnormality was detected in the DNA histogram. Each bar in the photomicrographs represents 200 *μ*m.

*Results of DNA content analysis* DNA content analysis was performed for 99 cases of dysplasia as listed in Table 2, and the DNA histograms were categorised as ‘abnormal’ or ‘no abnormality detected’ according to the criteria described in the Materials and Methods section. The five cases of squamous papilloma that were used as reference cases had no detectable abnormality in DNA content (Figure 2A). In all, 28 of the 99 dysplasia cases showed abnormal DNA content. Twenty-two of these dysplasias (79%) have progressed to carcinoma, and six have not progressed. There was a predominance of tongue lesions among dysplasias with abnormal DNA content, as 21 of the 28 cases with abnormal DNA were from the tongue. The histological grade of the dysplasias with abnormal DNA content ranged from mild (7 cases) to moderate (13 cases) to severe (8 cases).

A commonly observed pattern of abnormal cellular DNA content as seen in the DNA histogram had a large peak with DI between 1.7 and 2.3 (peritetraploid peak), along with aneuploid nuclei with DI >2.3 (Figure 2B). The peritetraploid peak greatly exceeded the peak formed by diploid cells in G2 and M phases of the cell cycle, which occupied a similar range of DI. Other cases show an aneuploid peak with DI between 1.15 and 1.7, along with aneuploid nuclei with DI >2.3 (Figure 2C). For descriptive purposes, we referred to the abnormal peak with DI between 1.15 and 1.7 as a hyperdiploid peak.

Seventy-one dysplasias were classified as having no detected abnormality in DNA content. Twenty-seven of these (38%) have progressed to carcinoma (Figure 2D).

*Association between abnormal DNA content and clinicopathological features* There was an association between grade of dysplasia and abnormal DNA content. Of 50 patients with mild dysplasia, 7 (14%) had abnormal DNA content, compared with 13 of 34 (38%) for moderate dysplasia, and 8 of 15 (53%) for severe dysplasia/carcinoma *in situ*. The trend was statistically significant with *P*=0.001 (exact Cochran–Armitage trend test).

To explore whether there was a higher frequency of abnormal DNA content among dysplasias from the tongue, we regrouped the sites of dysplasia into three groups: tongue, floor of mouth and others (buccal mucosa, gingiva–alveolar mucosa, soft palate–tonsillar pillar). Abnormal DNA content was found for 21 of 41 (51%) dysplasias from the tongue, 4 of 25 (16%) from floor of mouth and 3 of 33 (9%) for the others. The difference in frequency could not be explained by a higher proportion of moderate or severe dysplasia/carcinoma *in situ* in tongue lesions. Among tongue dysplasias, 56% were mild, 34% were moderate and 10% severe/carcinoma *in situ*. For floor of mouth dysplasias, 52% were mild, 32% moderate and 16% severe/carcinoma *in situ*. For buccal mucosa, gingiva–alveolar mucosa, soft palate–tonsillar pillar combined, 42% were mild dysplasias, 36% moderate and 21% severe/carcinoma *in situ*. The association between site of dysplasia and abnormal DNA content was statistically significant with *P*<0.0001 using Fisher's exact test.

**Figure 3:**
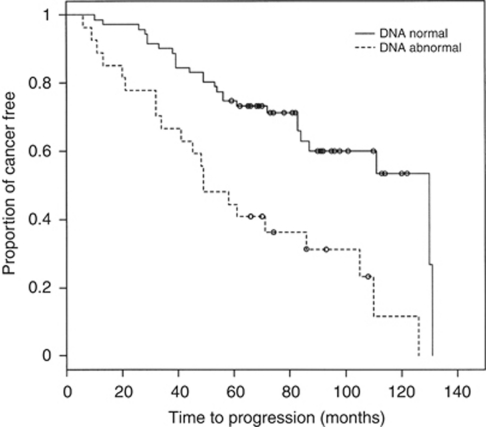
Kaplan–Meier plot of time to progression for patients with normal (*n*=71) *vs* abnormal (*n*=28) DNA histogram. Each ‘○’ indicates a censor, when the patient was last examined without evidence of progression to cancer. The difference in time to progression between the two groups was statistically significant (*P*=0.0001 by log-rank test).

We compared the two groups of patients with abnormal DNA histogram (*n*=28) and normal DNA histogram (*n*=71), with respect to time to progression to carcinoma. The median time to progression for patients with abnormal DNA histogram was 49 months (95% confidence interval (CI): 34–86 months), compared with 130 months (95% CI: 84–131 months) for patients with no detected abnormality on the DNA histogram. The time to progression plots are shown in Figure 3. The difference in time to progression between the two groups was statistically significant with *P*=0.0001 using log-rank test. All patients who did not progress to carcinoma were followed up for at least 5 years. The proportion of patients who were cancer free at 5 years were 75% (95% CI: 65–85%) for those with normal DNA histogram and 44% for those with abnormal DNA histogram (95% CI: 29–68%).

**Figure 4:**
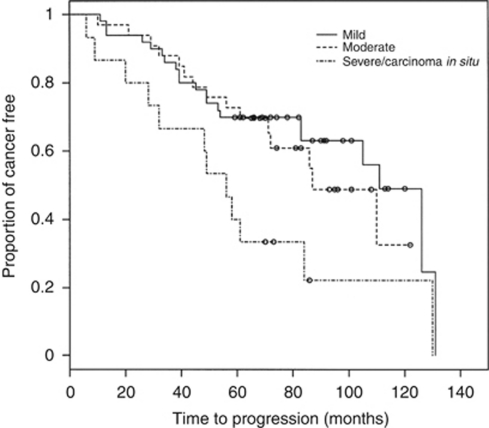
Kaplan–Meier plot of time to progression for patients with mild dysplasia (*n*=50), moderate dysplasia (*n*=34) and severe dysplasia/carcinoma *in situ* (*n*=15). Each ‘○’ indicates a censor, when the patient was last examined without evidence of progression to cancer. The differences in time to progression among the different grades were statistically significant (*P*=0.02, log-rank test).

We also examined the time to progression to carcinoma according to grade of dysplasia, as mild (*n*=50), moderate (*n*=34) and severe/carcinoma *in situ* (*n*=15). The last two groups were combined because of the small number of dysplasias in these groups. The median time to progression were 111 months (95% CI: 83–131 months), 87 months (95% CI: 71 months, upper limit not reached) and 56 months (95% CI: 32–84 months), respectively. The differences in time to progression to carcinoma among the three different grades were statistically significant (*P*=0.02, Figure 4).

To determine whether abnormal DNA content was a significant predictor of time to progression, corrected for the effect of site and grade, we performed multivariate analysis using Cox proportional hazard model. Abnormal DNA content was a significant predictor with *P*=0.003. The hazard ratio was 3.3 (95% CI: 1.5–7.4) for abnormal DNA content *vs* normal DNA content.

### Sequential samples of dysplasia and carcinoma

**Figure 5:**
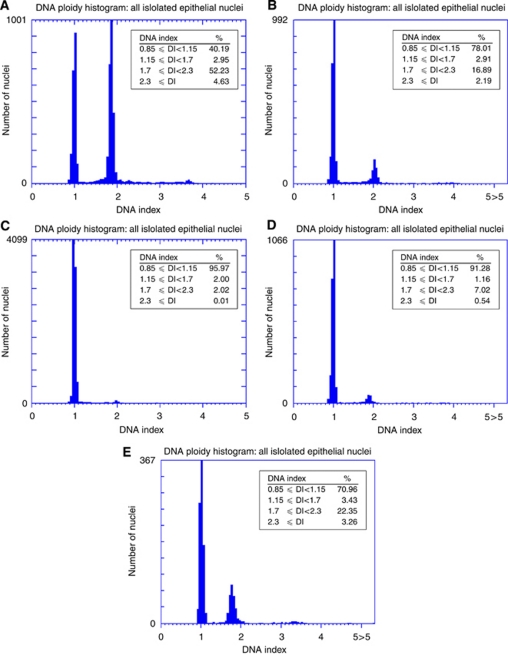
DNA histograms for sequential dysplasia and carcinoma. (**A**) Severe dysplasia of the tongue showing an abnormal peritetraploid peak with 52% of the nuclei and 4.5% of nuclei in the aneuploid region. (**B**) Carcinoma that developed 20 months later, with 17% of nuclei in a peritetraploid peak and 2.2% of nuclei in the aneuploid region. (**C**) Mild dysplasia of the tongue with no detected abnormality in the DNA histogram. (**D**) Carcinoma that developed 33 months later, with no detected abnormality in the DNA histogram. (**E**) Recurrent carcinoma in the neck that developed 12 months after treatment of the primary carcinoma, showing 22% of nuclei in a peritetraploid peak and 3.3% of nuclei in the aneuploid region.

For 15 of the dysplasias that progressed to carcinoma, it was possible to analyse the DNA content of sequential samples of dysplasia and carcinoma. Nine of these 15 dysplasias showed an abnormal DNA content. For 6 of these 9 cases, the subsequent carcinoma showed a similar pattern of DNA content abnormality as the corresponding dysplasia. However, the percentage of nuclei with abnormal DNA content was clearly lower in the carcinoma than in the preceding dysplasia in three cases (Figure 5A and B), whereas in the other three cases, the percentages remained similar. In three cases of dysplasia with abnormal DNA content, the large peritetraploid peak could not be detected in the subsequent carcinoma and the percentage of nuclei with DI >2.3 was also lower.

For five of six cases of dysplasia in which no abnormality was detected in DNA content, the carcinoma that followed also showed no significant abnormality in the DNA histogram. One of these carcinomas recurred as a neck mass, which showed a large peritetraploid peak in the DNA histogram (Figure 5C–E). In one case of dysplasia with no abnormality detected in DNA content, the patient developed carcinoma 131 months following the biopsy of dysplasia. Owing to the extent of the clinical white lesion, the carcinoma was treated with radiation and tumour tissue was not available for DNA content analysis. At 50 months following the first carcinoma, the patient developed a carcinoma at the anterior aspect of the white patch. The second carcinoma showed a large peritetraploid peak, along with many aneuploid nuclei with DI >2.3.

### Immunohistochemical staining for p53

**Figure 6:**
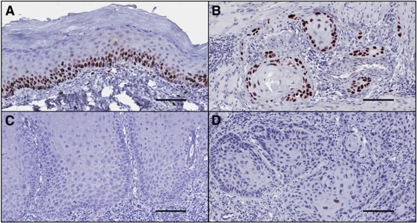
Immunohistochemical staining for p53 protein in sequential dysplasia and carcinoma, showing concordance of staining results for the dysplasia and subsequent carcinoma in two representative cases (counterstained with haematoxylin). (**A**) Moderate dysplasia of tongue showing moderate to strong staining of 10–50% of epithelial nuclei (grade 2 staining). (**B**) Carcinoma that developed 86 months later showing moderate to strong staining in >10% of epithelial nuclei (grade 2–3 staining). (**C**) Severe dysplasia of the tongue that showed no p53 staining. (**D**) Carcinoma that developed 20 months later also showed no p53 staining. Each bar in the photomicrographs represents 100 *μ*m.

We studied the expression of p53 protein in the samples of dysplasia and carcinoma where there was sufficient tissue to allow immunohistochemical staining of serial sections from tissue blocks used for DNA content analysis (77 of 99 cases stained). The purpose was to determine if p53 immunohistochemical staining could be related to abnormal DNA content. p53 staining was assessed on a semiquantitative scale from 0 to 3, as described in the Materials and Methods. For comparison of staining among groups of dysplasia, grades 0 and 1 staining were combined as ‘weak’ staining, whereas grades 2 and 3 were combined as ‘strong’ staining. Among 27 cases of dysplasia with abnormal DNA content, 12 cases (44%) showed strong p53 staining and 15 (56%) cases showed weak staining (Table 3, Figure 6A and C). Among 50 cases with no detected abnormality in DNA content, 19 (38%) exhibited strong staining and 31 (62%) were weak. The results did not demonstrate a higher proportion of strong p53 staining among cases with abnormal DNA content compared with cases with no abnormality in DNA content (44 *vs* 38%).

For 11 cases of sequential dysplasia and carcinoma, tissue was available for immunohistochemical staining for p53 for both dysplasia and carcinoma. In 10 of the 11 cases there was concordance of staining results for the dysplasia and subsequent carcinoma, supporting the notion that the carcinoma may have developed from the dysplasia (Figure 6).

## Discussion

The progression of epithelial dysplasia to carcinoma is not well understood at present. Longitudinal studies are needed to determine the frequency of progression of epithelial dysplasia to carcinoma and to identify biological markers that can predict disease progression, but relatively few such studies have been carried out (Schepman *et al*, 1998; Reibel, 2003; Holmstrup *et al*, 2006). A recent series of reports of long-term follow-up of oral leukoplakia and erythroplakia and the value of DNA content analysis as a prognostic marker have been retracted because of inaccuracies in the data (Curfman *et al*, 2006). Our retrospective study provided information on progression to carcinoma in a large cohort of patients who had one or more biopsies of epithelial dysplasia in an outpatient biopsy service. We showed that 8% of lesions (114 biopsies out of 1477) diagnosed as epithelial dysplasia progressed to carcinoma, after an interval of 6 months to 131 months. This represents the lower limit of the frequency of progression to carcinoma, because more carcinomas will likely develop with longer follow-up. There was a trend towards more cases of subsequent or concurrent carcinoma with increasing histological grade of dysplasia, but we noted that 37 of the 114 cases of subsequent carcinoma were found with mild dysplasia (Table 1). For these cases, histological assessment was not an accurate indicator of the risk of progression. There is therefore a need for biological markers to aid in the prediction of progression of dysplasia to carcinoma.

The data on progression of dysplasia to carcinoma should also be considered in the context of incidence of oral cancer and the proportion of oral cancer cases that are preceded by dysplastic lesions. Retrospective analysis of the files of the OPDS at the University of Toronto from 1993 to 2002 revealed 1477 cases of dysplasia, of which 158 cases were found to be associated with subsequent or concurrent carcinoma (Table 1). During the same 10-year period, the Oral Pathology Service at the University of Western Ontario received 1378 biopsies that were diagnosed as dysplasia or carcinoma *in situ* (T Daley, University of Western Ontario, London, ON, Canada, personal communication). In addition, we estimated that a similar number of biopsies of OEDs would be sent to pathology laboratories in hospitals across the province of Ontario, giving a total of approximately 4200 cases of OED in Ontario. Using the proportion of 158 of 1477 cases of dysplasia that were associated with oral cancer, we estimated that there were 450 cases of dysplasia that were associated with subsequent or concurrent carcinoma. For comparison, the Ontario Cancer Registry had 5120 cases of oral cancer in this 10-year period. The proportion of oral cancer cases that were preceded by a dysplastic lesion was likely higher than these figures would suggest. As dysplastic oral lesions are typically asymptomatic, they may remain undiagnosed in patients who do not have the benefit of regular oral examinations accompanied by biopsy of suspicious lesions. Our data suggested that improvements in the diagnosis and risk assessment of OED would assist the early diagnosis of oral cancer.

We showed that archival, formalin-fixed, paraffin-embedded biopsy specimens that have been in storage for as long as 15 years can be used successfully for DNA content analysis by image cytometry. Factors that limited the use of archival specimens included small size of the biopsy and inability to produce an adequate preparation of nuclei for image cytometry, but these affected only a minority of the cases we wished to study. The ability to use archival dysplasia specimens allowed us to relate the findings of DNA content analysis to long-term clinical outcome regarding progression to carcinoma.

Thick tissue sections (50 *μ*m) were used for preparation of nuclei, to decrease the likelihood of partial nuclei that would cause an artefactually lowered DNA content. It was not possible to perform microdissection of these thick sections to isolate epithelial nuclei from the nuclei of fibroblasts, endothelial cells and other non-epithelial cells. We divided nuclei into four groups of DI values and used previously published criteria to classify the DNA histogram as ‘abnormal’ or ‘no abnormality detected’ (see Materials and Methods).

We found a strong association between abnormal DNA content and progression to carcinoma. Of the 28 cases of dysplasia with abnormal DNA content, 22 were associated with subsequent carcinoma. Among the six patients with dysplasias that showed abnormal DNA content but have not progressed to carcinoma, one has a persistent dysplastic lesion, and three patients had re-excision of the dysplastic lesion followed by resolution. The remaining two patients’ lesions resolved after the first biopsy. Owing to the limited amount of clinical information that was available in our retrospective study, we could not analyse in detail the effect of surgical excision on progression of epithelial dysplasia, but we considered it possible that differences in surgical treatment could be a confounding factor in the association between abnormal DNA content and progression to carcinoma. In a multivariate analysis of DNA content, site and grade of dysplasia as predictors of time to progression, abnormal DNA content was a significant predictor with a hazard ratio of 3.3 (95% CI: 1.5–7.4). A prospective, long-term follow-up study of a large series of OED from multiple centres that collects information on the surgical treatment rendered for the dysplastic lesion, with conventional histological assessment and DNA content analysis by image cytometry, should provide further information on abnormal DNA content as a marker for progression to carcinoma. This line of investigation should improve our ability to identify and treat a subset of high-risk dysplastic lesions.

Allelic loss or LOH at microsatellite markers have been found in longitudinal studies to indicate the risk of progression of dysplastic oral lesions to carcinoma (Partridge *et al*, 2000; Rosin *et al*, 2000). In one study, LOH at 3p and/or 9p, with or without additional losses on 4q, 8p, 11q or 17p was predictive of progression. The proportion of dysplasias that did not progress at 5 years after diagnosis was 98% for cases without LOH at 3p and 9p, 63% for cases with LOH at 3p and/or 9p, and 53% for cases with LOH at 3p and/or 9p plus any of the other four arms (Rosin *et al*, 2000). In this study of DNA ploidy, the proportion who were cancer free at 5 years were 75% for cases with normal DNA histogram and 44% for cases with abnormal DNA histogram (see Results). It should be noted that the LOH study differed from our study in that many of the patients were followed up for <5 years. Neither LOH nor DNA ploidy analysis could identify all cases of dysplasia that progressed to carcinoma. Prospective studies using both LOH and DNA ploidy would be useful to determine whether these markers identify the same or complementary subsets of dysplasias that progress to carcinoma.

Our finding of a strong association between abnormal DNA content and progression to carcinoma agreed with a previous study of DNA ploidy analysis and malignant progression of OEDs, where 14 of 19 aneuploid dysplasias progressed to carcinoma (Torres-Rendon *et al*, 2009). In addition, we demonstrated for the first time that progressed dysplasias were often characterised by a high percentage of peritetraploid nuclei along with a smaller number of aneuploid nuclei (Figure 2B). Interestingly, our study of sequential cases of dysplasia and carcinoma showed that the large aberrations in DNA content seen in dysplasias often appeared reduced in the subsequent carcinomas (Figure 5A and B). The large peritetraploid peak that was seen in many DNA histograms of progressed dysplasias was rarely found in carcinomas. We were careful to select areas of carcinoma with a high carcinoma/stroma ratio for preparation of nuclei, so that the reduction in severity of DNA content abnormality was unlikely to be an artefact of dilution by stromal cells. Our observations suggested that epithelial cells with large aberrations in DNA content, which predominated in the dysplasia, have been replaced in the subsequent carcinoma by cells with less severe aberrations in DNA content. Studies of cell lines have shown that tetraploid cell populations are unstable intermediates in the development of aneuploidy (Ganem *et al*, 2007; Storchova and Kuffer, 2008). DNA content analyses of sequential biopsies of Barrett's oesophagus have shown that G2/tetraploid populations were intermediates in the progression to aneuploidy and carcinoma and that they were associated with loss of p53 (Galipeau *et al*, 1996; Maley *et al*, 2004). Studies of precancerous cervical lesions showed that tetraploidy occurred during the early stages of cervical carcinogenesis and preceded the development of aneuploidy (Olaharski *et al*, 2006). Although our study was limited by the small number of sequential samples and possible heterogeneity of DNA ploidy status within large lesions of carcinoma (Diwakar *et al*, 2005), our findings support the hypothesis that epithelial cell populations with grossly abnormal DNA content act as intermediates in the multistep process of oral carcinoma development. Further studies are needed to confirm this hypothesis. Dysplastic lesions with abnormal DNA content are at risk for progression to carcinoma, but they may also be susceptible to control by targeted therapy such as reactivation or augmentation of the DNA damage response (Storchova and Kuffer, 2008).

We hypothesised that abnormal DNA content in OED would be associated with loss of p53 function. In studies of Barrett's oesophagus, increase in the fraction of G2/tetraploid cells was associated with 17p allelic loss and p53 mutation (Galipeau *et al*, 1996). Increased immunohistochemical staining for p53 protein was reported to be associated with increased genetic instability in pre-neoplastic epithelium as measured by chromosomal *in situ* hybridisation (Shin *et al*, 2001). We therefore studied the expression of p53 protein by immunohistochemical staining, in parallel with DNA content analysis in 77 of the 99 cases of dysplasia. The immunohistochemical staining did not demonstrate an association between increased p53 staining and abnormal DNA content. Although increased p53 staining is often indicative of a mis-sense mutation in p53 resulting in a stabilised mutant protein, there are other types of p53 mutation, which lead to decreased or loss of staining. Sequencing of the p53 gene and analysis of deletion at the 17p locus would provide more definitive information on p53 function, but these investigations were not possible in our study due to insufficient tissue.

In all, 27 of the 49 progressed dysplasias in our study did not show abnormality in DNA content by image cytometry (Table 2). The DNA content histogram for these lesions appeared similar to the reference cases of squamous papilloma (Figure 2D). In our limited analysis of sequential dysplasia and carcinoma, five of six cases of progressed dysplasias with no abnormality in DNA content were associated with carcinoma with a similar DNA histogram (Figure 5C and D). This suggested that progression of dysplasia to carcinoma may not involve gross aberrations of DNA content, which are demonstrated by DNA image cytometry. Thus, there are at least two subsets of progressing dysplasias that differ in genetic characteristics. Combined analyses of OED that include DNA image cytometry and multiple genetic and epigenetic markers will be needed to identify a panel of biomarkers that will accurately predict the risk of carcinoma.

## Conclusions

Our retrospective study of OED showed that at least 8% of these lesions were associated with subsequent carcinoma. A subset (45%) of dysplasias which ultimately progressed were characterised by abnormal DNA content by DNA image cytometry. There was an association between abnormal DNA content and increased risk of progression of epithelial dysplasia to carcinoma. There is a need to identify additional biomarkers besides abnormal DNA content in order to accurately predict the progression from dysplasia to carcinoma.

## Figures and Tables

**Table 1:** Association between dysplasia and carcinoma, by histological grade and oral site

	**Number of biopsies diagnosed as dysplasia**	**Number of dysplasia with subsequent carcinoma (%)** a	**Number of dysplasia concurrent with carcinoma (%)** b	**Number with subsequent or concurrent carcinoma (%)**
All histological grades	1477	114 (7.7)	44 (3.0)	158 (10.7)
Mild	959	37 (3.9)	10 (1.0)	47 (4.9)
Moderate	326	34 (10.4)	9 (2.8)	43 (13.2)
Severe	149	35 (23.5)	14 (9.4)	49 (32.9)
Carcinoma *in situ*	43	8 (18.6)	11 (25.6)	19 (44.2)
All oral sites	1477	114 (7.8)	44 (3.0)	158 (10.7)
Tongue	271	36 (13.3)	10 (3.7)	46 (17.0)
Floor of mouth	329	39 (11.9)	15 (4.6)	54 (16.4)
Buccal mucosa	216	10 (4.6)	4 (1.9)	14 (6.5)
Gingiva	254	19 (7.5)	7 (2.8)	26 (10.2)
Palate, tonsillar pillar	224	7 (3.1)	3 (1.3)	10 (4.5)
Lip	114	1 (0.9)	4 (3.5)	5 (4.4)
Site not stated	69	2	1	3

a Carcinoma followed dysplasia by ⩾6 months.

b Dysplasia and carcinoma occurred within 6 months.

**Table 2:** Characteristics of the two groups of dysplasia used for DNA content analysis

	**Progressed to cancer**	**Did not progress**
*N*	49	50
		
*Age (years)*		
Mean (s.d.)	60 (13)	55 (14)^a^
Median (range)	60 (32–83)	55 (17–79)
		
*Sex*		
F (%)	22 (45)	25 (50)
M (%)	27 (55)	25 (50)
		
*Grade*		
Mild (%)	21 (43)	29 (58)
Moderate (%)	16 (33)	18 (36)
Severe (%)	11 (22)	3 (6)
Carcinoma *in situ* (%)	1 (2)	0 (0)
		
*Site*		
Tongue (%)	22 (45)	19 (38)
Floor of mouth (%)	14 (29)	11 (22)
Buccal mucosa (%)	5 (10)	8 (16)
Gingiva/alveolar mucosa (%)	4 (8)	9 (18)
Soft palate/tonsillar pillar (%)	4 (8)	3 (6)
Interval between dysplasia and carcinoma, median (range) in months	49 (6–131)	N/A
Duration of follow-up, median (range) in months	N/A	80 (59–122)
Abnormal DNA content (%)	22 (45)	6 (12)

Abbreviations: F=female; M=male; N/A=not applicable.

a The age of four patients in this group was not available.

**Table 3:** Results of p53 staining according to DNA content and progression to carcinoma

	**Number of cases with strong p53 staining (%)**	**Number of cases with none or weak p53 staining (%)**
Abnormal DNA content – 27 cases	12 (44)	15 (56)
Progressed to carcinoma	11	11
Have not progressed to carcinoma	1	4
No abnormality detected in DNA content – 50 cases	19 (38)	31 (62)
Progressed to carcinoma	11	6
Have not progressed to carcinoma	8	25
